# Respiratory Syncytial Virus Infection and Apnea Risk As Criteria for Hospitalization in Full Term Healthy Infants

**DOI:** 10.7759/cureus.53845

**Published:** 2024-02-08

**Authors:** Dyana Picache, Diana Gluskin, Asif Noor, Brooke Senken, Theresa Fiorito, Meredith Akerman, Leonard R Krilov, Jill Leavens-Maurer

**Affiliations:** 1 Pediatrics, NYU Grossman Long Island School of Medicine, Mineola, USA; 2 Hospital Medicine, Hackensack Meridian Ocean Medical Center, Neptune Township, USA; 3 Pediatric Infectious Diseases, NYU Grossman Long Island School of Medicine, Mineola, USA; 4 Pediatric Emergency Medicine, Indiana University School of Medicine, Indianapolis, USA; 5 Biostatistics, NYU Grossman Long Island School of Medicine, Mineola, USA

**Keywords:** pediatric emergency medicine, pediatric hospital medicine, acute bronchiolitis, apnea, respiratory syncytial virus (rsv)

## Abstract

Introduction

Apnea is recognized as a serious and potentially life-threatening complication associated with Respiratory Syncope Virus (RSV). The literature reports a wide range of apnea rates for infants with comorbid factors. Prematurity and young chronological age have been historically associated with the risk of apnea in hospitalized infants. Few studies have specifically examined the risk of apnea in healthy infants presenting to the emergency department.

Methods

This is a retrospective review of infants diagnosed with RSV using a PCR assay. Patients were divided into "mild" and "severe" cohorts based on symptoms at presentation. This study occurred in the NYU Langone Long Island (NYULI) pediatric emergency department (ED), a midsize academic hospital in the Northeast United States. The study included infants <6 months of age, born full term without comorbid conditions such as chronic lung or cardiac conditions, seen in NYULI ED over three consecutive RSV seasons (2017-2020). The primary outcome was the risk of apneic events. Secondary outcomes included hospital admission, ICU admission, length of stay, and supplemental oxygen support.

Results

The risk of apnea was <2%, regardless of disease severity. There were no significant differences in demographics between mild and severe disease. Cohorts differed significantly in the number of hospitalizations (41 milds vs. 132 severe), ICU admissions (2 milds vs. 27 severe), need for oxygen support (17 milds vs. 92 severe), hospital readmissions (2 milds vs. 42 severe), and length of stay (2 days milds vs. 3 days severe).

Conclusions

Apnea does not pose a significant risk for healthy full-term infants with RSV disease of any severity. The decision to admit this population to the hospital should be based on clinical presentation and not solely on the perceived risk of apnea.

## Introduction

Respiratory Syncytial Virus (RSV) is the leading cause of respiratory tract illness in infants and accounts for 3.4 million hospitalizations annually worldwide [1,2]. The reported risk of apnea with RSV varies widely in the literature from 1.6% to 20%, with studies including variations in clinical settings (inpatient versus outpatient) and a spectrum of comorbidities [3-6]. 

Historically, most literature regarding RSV focused on infants with high-risk conditions for apnea, such as prematurity, airway anomalies, chronic lung disease, congenital heart disease, and neuromuscular disease, especially during the development of palivizumab [7-10]. More recent studies report a low rate of apnea (<1%) in hospitalized full-term infants without comorbidities; however, there is a lack of literature that examines this population [11].

Admission rates for infants with bronchiolitis have increased over the past two decades, likely due to several advancements in diagnostic technology, such as pulse oximetry and PCR-based respiratory viral panels (RVP) [12-15]. Although this technology allows for more specific diagnoses, it may lead to unnecessary hospitalizations from the anticipation of worsening clinical course in healthy full-term infants, which may lead to inconsistent practices based on providers' personal experiences. The American Academy of Pediatrics Clinical Practice Guidelines (2014) do not provide emergency department (ED) disposition recommendations for healthy young infants without severe RSV disease [16]. It is worth exploring objective measures that would warrant hospitalization, given that RSV-related admissions are associated with a high economic burden as well as indirect social impacts such as caregiver absence from work [17].

We hypothesize an overall low risk of apnea in full-term infants with RSV disease regardless of severity at presentation. We sought to study the risk of apnea in full-term infants <6 months of age presenting to the ED with a positive RSV test comparing mild versus severe disease.

This article was previously presented at the Pediatric Academic Societies Conference on April 23, 2022.

## Materials and methods

The study received approval by the NYU Langone Health institutional review board, study number S19-00561. This research received no specific grant from any agency in the public, commercial, or not-for-profit sectors. 

A retrospective chart review of infants evaluated in the pediatric ED was conducted at NYU Langone Hospital Long Island (NYULI), a midsize academic center in the Northeast that has the capability of delivering high-flow nasal cannula (HFNC) on the regular pediatric floor. Data were collected using two electronic medical record (EMR) systems, Soarian and EPIC. Cases from RSV seasons 2017-2018, 2018-2019, and 2019-2020 were selected based on ICD-10 codes of B97.4, Respiratory Syncytial Virus, and J21.0, Acute Bronchiolitis.

**Figure 1:**
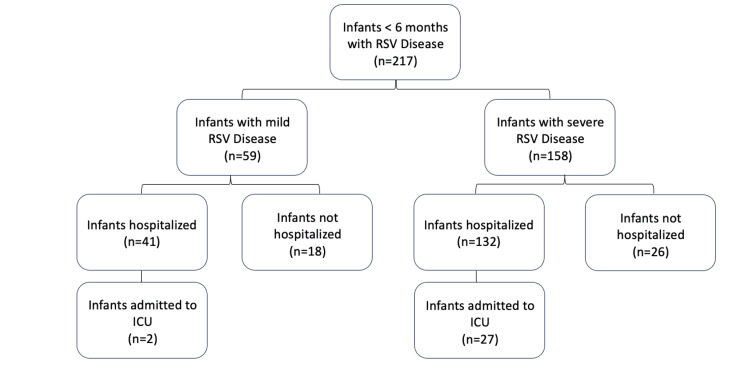
RSV-related hospitalizations in infants < 6 months RSV: Respiratory Syncytial Virus

Inclusion criteria were infants aged 0-6 months diagnosed with RSV using PCR RVP. Infants with high-risk conditions, including a history of prematurity (≤ 37 weeks), prior hospitalization for apnea, chronic lung disease, congenital heart disease, airway anomalies, neuromuscular disease, or genetic disorder (e.g., Trisomy 21, cystic fibrosis) were excluded from the study (Figure 1). Demographic data were collected, including age, sex, race, birth weight, number of days with symptoms, and environmental factors (smoking, daycare, breastfeeding).

Apnea was defined as cessation of breathing for 20 seconds, bradycardia <50bpm, or color change as reported by parents or medical providers at presentation to the ED and during the hospital course.

Infants were classified into "mild" or "severe" RSV disease groups using a modified predictive model for the need for escalated care proposed by Freire et al. [18]. We chose this predictive model as it provides early risk stratification, specifically in the ED setting. These clinical variables included respiratory rate ≥ 60 breaths per minute, Retractions, Oxygen saturation <90% in room air, Poor oral intake as reported by parents, and Dehydration as recorded by the healthcare provider, including dry mucous membranes and prolonged capillary refill.

If subjects had one or more clinical variables, they were classified as "severe". If the subject had none of these clinical variables, they were classified as "mild". The study population was also stratified into groups by age in months, < 1 month, ≥1 month and ≤ 3 months, > 3 months, and ≤ 6 months.

The primary outcome was to compare the risk of apnea based on RSV disease severity and age. Secondary outcomes consisted of variations in clinical course, including length of stay (LOS), oxygen requirement, intensive care (ICU) admission, and readmission rate.

Descriptive statistics (median [25th, 75th percentiles] for continuous variables; frequencies and percentages for categorical variables) were calculated separately by group (Mild vs. Severe RSV disease). As deemed appropriate, the groups were compared using the chi-square test or Fisher's exact test for categorical variables and the Mann-Whitney test, the non-parametric counterpart to the two-sample t-test, for continuous data. The Shapiro-Wilk test was used to test the normality of the continuous parameters. Due to the skewness of the data and the smaller sample size, a non-parametric test was used to compare the groups. Additional analyses were performed by age (<1 month, 1 ≤ age ≤ 3 months, and 3 < age ≤ 6 months). The Kruskal-Wallis test was used to compare these 3 groups for continuous data.

Standard methods of survival analysis were applied to LOS data. All subjects were "discharged alive" and therefore, no data was considered censored. Kaplan-Meier/Product-Limit Estimates and their corresponding 95% confidence intervals were computed using Greenwood's formula to calculate the standard error. Additionally, Kaplan-Meier product limit curves were computed, where the group was used as the stratification variable, and the groups were compared using the log-rank test.

A result was considered statistically significant at the p<0.05 level of significance. All analyses were performed using SAS version 9.4 (SAS Institute Inc., Cary, NC).

## Results

A total of 217 infants met the study criteria. There were 59 infants with mild RSV disease and 158 with severe. The groups were similar in age, sex, and ethnicity (Table 1). There was no difference in environmental factors such as smoking, daycare, or breastfeeding between the two severities. 

**Table 1:** Patient Characteristics by Severity *Data reported as mean ± standard deviation (median); categorical variables presented as n (%).

	Mild RSV disease (n=59)	Severe RSV disease (n=158)	p-value
Patient Age (months)	2.4 (1.2, 4.8)	2.4 (1.2, 3.6)	0.9716
Birth weight (kg)	3.29 (3.02, 3.60)	3.28 (3.06, 3.67)	0.4256
Day of illness at time of presentation	3 (2, 5)	3 (2, 4)	0.6338
Respiratory Rate at presentation	37 (32, 40)	40 (32, 48)	0.0095
Oxygen Saturation at presentation	98 (97, 100)	97 (94, 98)	<0.0001
Sex			
Male	34 (57%)	88 (55%)	0.7986
Female	25 (42%)	70 (44%)	
Ethnicity			
White	23 (38%)	72 (45%)	0.2802
Black	6 (10%)	20 (12%)	
Hispanic	27 (45%)	49 (31%)	
Asian	1 (1.6%)	9 (5.7%)	
Unknown	2 (3.3%)	8 (5.0%)	
Smoking	1 (1.7%)	8 (5.2%)	0.4495
Daycare	5 (10%)	6 (4.8%)	0.2981
Breastfed	21 (36%)	57 (37%)	0.9042
Respiratory Rate ≥ 60	0 (0%)	13 (8.3%)	0.0217
Retractions at presentation	0 (0%)	100 (63%)	<0.0001
Oxygen Saturation < 90	0 (0%)	11 (7%)	0.0378
Oral intake poor	0 (0%)	106 (68%)	<0.0001
Dehydration present	0 (0%)	26 (16%)	0.0008
Bradycardia	0 (0%)	2 (1.2%)	1.0000
Hypoxia	8 (13%)	56 (35%)	0.0017
Color Change	3 (5%)	4 (2.5%)	0.3944

Younger infants were found to have high respiratory rates and lower oxygen saturation in room air (Table 2). Additionally, infants < 1 month presented to the emergency room earlier compared to infants 3 < age ≤ 6 months. Among environmental factors, increased daycare attendance and decreased breastfeeding were found in older infants.

**Table 2:** Patient Characteristics by Age *Data reported as mean ± standard deviation (median); categorical variables presented as n (%).

	< 1 month (n = 12)	1 ≤ age ≤ 3 months (n = 129)	3 < age ≤ 6 months (n = 76)	p-value
Patient Age (months)	0.53 (0.28, 0.8)	1.48 (1.2, 2.4)	4.8 (3.6, 6)	<0.0001
Birth weight (kg)	3.33 (3.01, 3.8)	3.32 (3.05, 3.62)	3.26 (3.04, 3.62)	0.9506
Day of illness at time of presentation	2.5 (1.5, 3)	3 (2, 4)	4 (2, 5)	0.0314
Respiratory Rate at presentation	45 (36, 50)	40 (34, 48)	35 (30, 41)	0.0004
Oxygen Saturation at presentation	93.5 (89.5, 96)	97 (95, 99)	97 (95, 99)	0.0018
Sex				
Male	7 (58%)	70 (54%)	45 (59%)	0.7794
Female	5 (41%)	59 (45%)	31 (41%)	
Ethnicity				
White	5 (42%)	58 (45%)	32 (42%)	0.6244
Black	2 (17%)	15 (12%)	9 (12%)	
Hispanic	3 (25%)	41 (32%)	32 (42%)	
Asian	1 (8%)	7 (5.4%)	2 (2.6%)	
Unknown	1 (8%)	8 (6.2%)	1 (1.3%)	
Smoking	0 (0%)	6 (4.8%)	3 (4%)	1.0000
Daycare	0 (0%)	3 (3%)	8 (13%)	0.0419
Breastfed	6 (55%)	55 (45%)	17 (23%)	0.0046
Respiratory Rate ≥ 60	1 (9%)	10 (7.8%)	2 (2.7%)	0.2847
Retractions at presentation	8 (67%)	63 (49%)	29 (38%)	0.1130
Oxygen Saturation< 90	3 (25%)	7 (5.5%)	1 (1.3%)	0.0100
Oral intake poor	6 (55%)	64 (51%)	36 (47%)	0.8485
Dehydration present	1 (9%)	14 (11%)	11 (14%)	0.7192
Bradycardia	0 (0%)	1 (0.8%)	1 (1.3%)	1.0000
Hypoxia	8 (67%)	43 (33%)	13 (17%)	0.0007
Color Change	0 (0%)	6 (4.7%)	1 (1.3%)	0.6180

When we examined the data based on clinical outcome, we found that Infants presenting with mild RSV infection had no episodes of apnea (0%), and those with severe infection had a low rate of apnea (1.9%) (Table 3). A statistically significant difference between severity scores was observed in hospital admissions, LOS, oxygen requirement, ICU admission, and hospital readmission. The majority of severe RSV cases received oxygen support compared to less than half of mild cases, and of the infants that received support, most severe cases required at least HFNC, while most mild cases required only a regular nasal cannula (Table 3). Twenty-seven percent of the severe cases required ICU-level care, and LOS was one day longer than mild cases. Forty-two severe cases were readmitted after initial discharge compared to two mild cases.

**Table 3:** Clinical outcomes by severity HFNC: high-flow nasal cannula; BiPAP: bi-level positive airway pressure; LOS: Length of stay

	Mild RSV disease (n=59)	Severe RSV disease (n=158)	p-value
Apnea	0 (0.0%)	3 (1.9%)	0.5643
Oxygen Requirement			
No	42 (71%)	66 (41%)	0.000443
Nasal Cannula	11 (18%)	33 (20%)	
HFNC	5 (8.4%)	41 (25%)	
BIPAP	1 (1.6%)	18 (11%)	
Hospital Admission	41 (69%)	132 (83%)	0.0220
ICU Admission	2 (3.3%)	27 (17%)	0.0083
Hospital Readmission	2 (3.8%)	42 (26%)	<0.0001
Hospital LOS, median days (95% CI)	2 (1, 3)	3 (2, 4)	0.0134

There was no significant difference in the risk of apnea when comparing age groups, with no apneic events in infants < 1 month and a similar rate between the 1 ≤ age ≤ 3 and 3 < age ≤ 6 groups (Table 4). The infants with apneic episodes were all less than 4 months old. All infants < 1 month were admitted, compared to 85% of 1 ≤ age ≤ 3 and 67% of 3 < age ≤ 6. The youngest subgroup required the highest rate of BIPAP and highest rate of ICU admission (42%). The hospital length of stay was highest, with a median of 7 days, in the 3 to 6 months group.

**Table 4:** Clinical outcomes by age HFNC: high-flow nasal cannula; BiPAP: bi-level positive airway pressure; LOS: Length of stay

	< 1 month (n = 12)	1 ≤ age ≤ 3 months (n = 129)	3 < age ≤ 6 months (n = 76)	p-value
Apnea	0 (0%)	2 (1.6%)	1 (1.3%)	1.0000
Oxygen Requirement				
No	2 (17%)	57 (44%)	49 (64%)	0.0046
Nasal Cannula	4 (33%)	26 (20%)	14 (18%)	
HFNC	3 (25%)	33 (26%)	10 (13%)	
BIPAP	3 (25%)	13 (10%)	3 (3.9%)	
Hospital Admission	12 (100%)	110 (85%)	51 (67%)	0.0015
ICU Admission	5 (42%)	20 (16%)	4 (5.3%)	0.0014
Hospital Readmission	3 (27%)	25 (20%)	16 (21%)	0.8196
Hospital LOS, median days (95% CI)	3 (2, 4)	1.5 (1, 2)	7 (2, 11)	<0.0001

## Discussion

We found a low incidence of apnea in healthy full-term infants ≤ 6 months, regardless of RSV disease severity or age. The incidence of apnea was 1.9% in the severe RSV disease, while no cases of apnea were identified in the mild disease group. When studying the occurrence of apnea based on age, there were no apnea cases in infants ≤ 1 month of age on presentation to ED or during hospital stay for those admitted.

Prior articles on apnea in RSV infection had focused on hospitalized premature infants, which preferentially examines more severe cases of the disease. In a 1977 systematic review of 13 studies, including 5,575 hospitalized infants and young children with RSV, the incidence of apnea ranged from a high of 23.8% to a low of 1.2%. Prematurity was identified as an independent risk factor, but chronological age was not always available. This review mainly included articles studying infants with neuromuscular disorders and previous history of apnea [3].

Our results support findings in more recent literature [11]. A 2009 systematic review questioned whether a full-term infant without other risk factors, such as prematurity or chronic lung disease, requires hospitalization. This review demonstrated a decline in the overall prevalence of apnea in recent studies when compared to studies from the 1970s in infants [11]. A retrospective review from 2006 included 691 hospitalized infants with bronchiolitis. There were no apneic events for infants assessed to be at a low risk as determined by age >1 month for full-term infants or 48 weeks postconceptional age for preterm infants and absence of any previous apneic event at presentation to the hospital [6]. Recent studies report a low rate of apnea < 1% in healthy, full-term infants, values consistent with our current study's findings [6,11,19].

While the majority of prior studies on apnea in RSV infection have thus far focused on hospitalized infants, we obtained our population from an ED setting, which allowed us to characterize infants with milder disease, a population that comprises the majority of RSV cases and, therefore may be more applicable to the general population. Another strength of the study is the sample of three RSV seasons considering seasonal variability of severity and prevalence.

Our study had several limitations, the first of which is the distribution of age groups. Our study only included 12 infants < 1 month, thereby restricting the examination of the group often considered the most at-risk for severe disease. By its design, our retrospective study may be limited by the subjectivity of presenting symptoms based on the provider's discretion. Additionally, our study took place in a single medical center, which may limit generalizability to other geographic locations and settings.

## Conclusions

Apnea is rare in healthy full-term infants with RSV disease of any severity and is not associated with a chronological age of infants. We conclude that hospitalization for these infants should not be based solely on the perceived or anticipated risk of apnea. Furthermore, differentiating mild vs severe disease may help predict clinical course and length of stay. Therefore, future studies are necessary to develop and validate a standard definition of disease severity. This study may be used to help guide management in a primary care or emergency room setting and may help ease the burden of unnecessary RSV bronchiolitis hospitalizations. This is especially important given strained healthcare resources in the light of a shifted RSV epidemiology since the COVID-19 pandemic in 2020.
